# Membranolytic Activity Profile of Nonyl 3,4-Dihydroxybenzoate: A New Anti-Biofilm Compound for the Treatment of Dermatophytosis

**DOI:** 10.3390/pharmaceutics15051402

**Published:** 2023-05-04

**Authors:** Caroline B. Costa-Orlandi, Níura M. Bila, Jean Lucas C. Bonatti, Carolina O. Vaso, Mariana B. Santos, Carlos R. Polaquini, Mariana M. Santoni Biasioli, Rondinelli D. Herculano, Luis O. Regasini, Ana Marisa Fusco-Almeida, Maria José S. Mendes-Giannini

**Affiliations:** 1 Department of Clinical Analysis, School of Pharmaceutical Sciences, São Paulo State University (U.N.E.S.P.), Araraquara 14800-903, SP, Brazil; 2 Department of Para-Clinic, School of Veterinary, Eduardo Modlane University (UEM), Maputo 257, Mozambique; 3 Department of Chemistry and Environmental Sciences, Institute of Biosciences, Humanities and Exact Sciences, São Paulo State University (U.N.E.S.P.), Sao Jose do Rio Preto 15054-000, SP, Brazil; 4 Department of Biological Sciences, School of Pharmaceutical Sciences, São Paulo State University (U.N.E.S.P.), Araraquara 14800-903, SP, Brazil; 5 Department of Bioprocesses and Biotechnology, School of Pharmaceutical Sciences, São Paulo State University (U.N.E.S.P.), Araraquara 14800-903, SP, Brazil

**Keywords:** dermatophytes, biofilms, anti-biofilm activity, antifungal drugs, mechanism of action, RT-PCR

## Abstract

The ability of dermatophytes to live in communities and resist antifungal drugs may explain treatment recurrence, especially in onychomycosis. Therefore, new molecules with reduced toxicity that target dermatophyte biofilms should be investigated. This study evaluated nonyl 3,4-dihydroxybenzoate (nonyl) susceptibility and mechanism of action on planktonic cells and biofilms of *T. rubrum* and *T. mentagrophytes*. Metabolic activities, ergosterol, and reactive oxygen species (ROS) were quantified, and the expression of genes encoding ergosterol was determined by real-time PCR. The effects on the biofilm structure were visualized using confocal electron microscopy, scanning electron microscopy (SEM), and transmission electron microscopy (TEM). *T. rubrum* and *T. mentagrophytes* biofilms were susceptible to nonyl and resistant to fluconazole, griseofulvin (all strains), and terbinafine (two strains). The SEM results revealed that nonyl groups seriously damaged the biofilms, whereas synthetic drugs caused little or no damage and, in some cases, stimulated the development of resistance structures. Confocal microscopy showed a drastic reduction in biofilm thickness, and transmission electron microscopy results indicated that the compound promoted the derangement and formation of pores in the plasma membrane. Biochemical and molecular assays indicated that fungal membrane ergosterol is a nonyl target. These findings show that nonyl 3,4-dihydroxybenzoate is a promising antifungal compound.

## 1. Introduction

Dermatophytosis is among the most prevalent mycoses worldwide, affecting a large proportion of the population regardless of age, immunological status, sex, or race [1,2,3,4]. The need for prolonged treatment, recurrence of infections, frequent reports of resistance, and the possibility of biofilm formation, especially in onychomycoses, are the main limitations for the treatment of this disease [5,6,7,8,9,10].

Azoles and allylamines are classically used to treat dermatophytosis and can be administered orally and/or topically, alone or in combination, depending on the severity of the infection and patient prognosis [7,11,12,13]. Terbinafine, fluconazole, and itraconazole are the most commonly used drugs for systemic treatment. Ketoconazole and griseofulvin are prescribed according to the clinical manifestations of dermatophytosis but are no longer recommended for the treatment of onychomycosis [11,14,15]. It is important to note that oral administration of fluconazole is not approved in some countries for the treatment of dermatophytic onychomycoses [15,16]. Despite their high efficacy, systemic antifungals should be used with caution because of the greater likelihood of adverse effects than topical administration, including interactions with other drugs, congestive heart failure, and hepatotoxicity [15].

Terbinafine, cyclopirox, and amorolfine are still widely used as topical treatments in some countries, while efinaconazole, tavaborol, and luliconazole have gained prominence owing to their safety and efficacy [7,17,18,19,20,21]. Topical therapy is generally nontoxic to patients because systemic absorption does not occur, minimizing the likelihood of interactions with other drugs and the emergence of severe adverse effects. Topical drug administration has disadvantages in the case of onychomycosis owing to low penetration into the nail plate caused by several physiological factors in addition to the composition of the pharmaceutical form. The cost of newer antifungals is high, making it difficult for many people to adhere to the treatment regimen for a long time [7,15,16].

Recurrence and reinfection, common in onychomycoses, may be related to biofilm formation and other factors [8,9,10,22]. Most infections caused by microorganisms are associated with the formation of sessile communities and three-dimensional structures covered by a complex polymeric extracellular matrix [23,24,25]. This phenotype promotes advantageous protection for microorganisms, which is provided by physical and genetic factors such as the barrier effect of biomass, microenvironment composition, concentration of metabolites and gases, and modification of gene regulation [25,26,27].

Fungal biofilms are difficult to treat and eradicate using currently available antifungals [28,29]. The dose of an antifungal agent required to eradicate certain fungal biofilms can exceed the recommended therapeutic concentration [28]. Therefore, novel compounds capable of eradicating fungal biofilms are being investigated.

Nonyl 3,4-dihydroxybenzoate (nonyl) is a synthetic derivative of protocatechuic acid. Protocatechuic acid and its derivatives have been studied and exhibit a broad spectrum of biological and antifungal activities [7,30,31,32,33,34,35]. Our group has described its fungicidal activity [30,35] and its effectiveness in reducing the metabolic activity and topography of dermatophyte biofilms [7]. However, further studies are needed to demonstrate the damage caused by this compound to the ultrastructure of biofilms compared to conventional antifungals, in addition to investigating its mechanism of action in fungal cells. Therefore, this study aimed to determine the susceptibility of mature biofilms and planktonic cells of *T. rubrum* and *T. mentagrophytes* to nonyl, compare these results to the activities of three conventional antifungal drugs, and study its mechanism of action in dermatophytes.

## 2. Materials and Methods

### 2.1. Dermatophytes Tested

Standard references (*T. rubrum* ATCC 28189 INCQS 40051 and *T. mentagrophytes* ATCC 11481 INCQS 40118) and clinical strains (*T. rubrum* 143 and *T. mentagrophytes* 66) were used. ATCC strains were obtained from the Clinical Mycology Laboratory of the School of Pharmaceutical Sciences at the Universidade Estadual Paulista, Araraquara, SP, Brazil. Clinical strains were kindly provided by Prof. Dr. Maria Aparecida Resende-Stoianoff from the Clinical Mycology Laboratory at the Federal University of Minas Gerais and isolated from patients with onychomycosis at the dermatology outpatient clinic of a tertiary care hospital. This study was published by Costa-Orlandi et al. [3] and approved by the Ethics Committees of the Federal University of Minas Gerais and Hospital Santa Casa de Misericordia de Belo Horizonte (Ethics 004.0.107.000-06/094-2009). The fungi were maintained in sterile distilled water, cultivated on potato dextrose agar (Difco, BD biosciences, Sparks, MD, USA) and incubated at 28 °C for seven days [36].

### 2.2. Synthesis of Nonyl 3,4-Dihydroxybenzoate

Nonyl protocatechuate was prepared as previously described by Faria et al. [37], Soares et al. [30], and Costa-Orlandi et al. [7]. Briefly, the synthesis was performed by standard esterification reactions using protocatechuic acid (Sigma-Aldrich, St. Louis, MO, USA) and nonyl alcohol, in *N*′,*N*′-dicyclohexylcarbodiimide (DCC) and *p*-dioxane. The residue was partitioned with ethyl acetate and filtered. The filtrate was washed with NaHCO_3_ solution and water and dried over MgSO_4_. The crude product was purified over a silica gel column (0.06–0.02 mm, A.C.R.O.S. Organics, Waltham, MA, USA) and eluted using CHCl_3_/CH_3_OH (98:2). The structure of the compound was confirmed using ^1^H and ^13^C Nuclear Magnetic Resonance spectroscopy. NMR spectra were recorded using a Bruker spectrometer (300 MHz, 7.1 T). The purity of the compound was determined by high-performance liquid chromatography with a photodiode array detector (HPLC-PAD) using the peak area. HPLC-PAD chromatograms were obtained on Agilent Technologies 1220 Infinity equipment, a photodiode array system (Agilent Technologies Model 1260 Infinity), and an Agilent Zorbaz Eclipse Plus C-18 column (250 mm × 4.6 mm, 5 µm) using methanol:water (98:2) as the mobile phase (1.0 mL/min). The NMR spectra and HPLC chromatograms are presented in the Supplementary Material (Figures S1–S3).

### 2.3. Preparation of Conventional Antifungals and Nonyl Protocatechuate (Nonyl 3,4-Dihydroxybenzoate)

Fluconazole, griseofulvin, and terbinafine were purchased from Sigma-Aldrich (St. Louis, MO, USA). Stock solutions of the antifungal drugs were prepared according to document M-38 A2 proposed by the Clinical and Laboratory Standards Institute (C.L.S.I.) [36]. Stock solutions of fluconazole were prepared in sterile distilled water at concentrations ranging from 0.625 mg/L to 5120 mg/L. For terbinafine and griseofulvin, stock solutions in DMSO were prepared at concentrations ranging from 0.1 to 3200 mg/L for the former and from 6.25 to 51,200 mg/L for the latter. Working solutions were prepared from the stock solutions in RPMI 1640 medium with glutamine, without sodium bicarbonate, and with phenol red as a pH indicator (Gibco Life Technologies, Grand Island, NY, USA), buffered with MOPS (Sigma-Aldrich, St. Louis, MO, USA). Working solutions of fluconazole and griseofulvin were prepared to obtain concentrations ranging from 0.0625 to 512 mg/L. For terbinafine, working solutions were prepared in the range of 0.001–32 mg/L. Nonyl was aseptically diluted in DMSO to obtain a DMSO concentration in the working solution of less than 1%. Working solutions were prepared at concentrations ranging from 0.015 to 500 mg/L.

### 2.4. Effects of Antifungal Drugs and Nonyl 3,4-Dihydroxybenzoate on T. rubrum and T. mentagrophytes, Planktonic Cells, and Mature Biofilms

One hundred microliters of the working solutions of the antifungals and the compound at twice the final desired concentration were added to 96-well microdilution plates to evaluate the effects of the antifungals and nonyl against planktonic cells. Inoculums were prepared in RPMI 1640 medium to reach a final concentration corresponding to 1 × 10^6^ cells/mL, and 100 µL of the fungal suspensions were added to each well along with their respective controls. The microplates were incubated at 37 °C for 72 h. According to our previously established protocol, biofilms were formed in 96-well plates with RPMI 1640 medium [10]. Biofilm susceptibility assays were conducted as described by Costa-Orlandi et al. [4,7]. Briefly, the culture medium was aspirated after maturation, and the wells were washed with 0.85% sterile saline. Then, 100 µL of each dilution of different concentrations of the antifungals and the compound were distributed in 96-well plates with their respective controls: the sterility control of the medium and the untreated biofilms. The microplates were incubated at 37 °C for 72 h. The XTT reduction assay was used to quantify the metabolic activities of planktonic cells and biofilms. The plates were read at 490 nm using a spectrophotometer (Microplate Reader iMarkTM; Bio-Rad, Hercules, CA, USA). Inhibition was defined as a reduction in metabolic activity of at least 50%.

### 2.5. Scanning Electron Microscopy (SEM) of Treated Biofilms

SEM was performed to visualize the effects of nonyl and antifungal drugs on fungal biofilm topography. Biofilms were formed in 24-well plates and treated with fluconazole (512 mg/L), griseofulvin (512 mg/L), terbinafine (32 mg/L), or nonyl (250 mg/L). The plates were incubated at 37 °C for 72 h. The samples were fixed and dried, and the bottoms of the plates were cut using a scalpel, according to Costa-Orlandi et al. [4,7,10], Bila et al. [20], Garcia et al. [38], and Vaso et al. [39]. The samples were fixed on carbon tape, mounted on aluminum cylinders with silver, and placed in a high-vacuum evaporator for gold plating. The topographies of the biofilms were analyzed using a scanning electron microscope (SEM; JEOL JSM-6610LV Peabody, MA, USA) at the School of Dentistry of the Sao Paulo State University (U.N.E.S.P.) Araraquara.

### 2.6. Confocal Microscopy of Treated Biofilms

Confocal microscopy was used to visualize the thickness of biofilms after antifungal treatment. Biofilms were grown in 24-well plates containing sterile round coverslips. After maturation, the culture medium was aspirated, and the biofilms were washed with sterile saline. Terbinafine (1 mL, 32 mg/L) and nonyl (250 mg/L) were then added. The plates were incubated for 72 h at 37 °C. The untreated biofilms were used as controls. A solution of C.A.A.F. (Concanavalin A—conjugated to Alexa Fluor 488—*Molecular Probes*, Eugene, OR, USA, 25 µM) and FUN 1 (Molecular Probes, USA, 10 µM) was prepared. This mixture was added to wells containing the treated and untreated biofilms, and the plates were incubated at 37 °C for 45 min while protected from light. Then the wells were washed, and the coverslips were removed, covered with 4 μL of Fluoromount-G (Sigma-Aldrich), and deposited on microscope slides for observation under a confocal microscope Leica TCS SP5 (Leica Microsystems, Wetzlar, Germany) from the School of Medicine of the University of Sao Paulo (U.S.P.) Ribeirão Preto. The images were processed using L.A.S. AF 1.8.2 build 1465 Leica Microsystems CMS Gmgh [4,10,40].

### 2.7. Determination of the Mechanism of Action of Nonyl

#### 2.7.1. Transmission Electron Microscopy

To verify the damage caused by the compounds to the fungal ultrastructure, 1 × 10^6^ cells/mL of *T. rubrum* ATCC 28189 were treated with low concentrations of nonyl (1 and 0.5 mg/L), fluconazole (64 mg/L), and amphotericin (1 mg/L) as a control. After the incubation period, the contents of the microdilution plate wells were transferred to 15-mL conical tubes, washed three times with phosphate-buffered saline (PBS, pH 7.2) three times after centrifugation, washed again, and fixed with 2.5% glutaraldehyde solution in sodium cacodylate buffer (Electron Microscopy Sciences, Fisher Scientific, Waltham, MA, USA). After fixation, cells were washed and exposed to potassium dichromate and uranyl acetate. The fungal elements were washed, soaked in agar, and cut into small cubes (approximately 0.5 to 1 mm^3^), which were dehydrated using increasing concentrations of ethanol. The samples were then placed in Spurr resin, sectioned using an ultramicrotome, and observed under a Jeol JEM-100 CXII transmission electron microscope equipped with a Hamamatsu ORCA-HR digital camera at the School of Medicine of the University of Sao Paulo (U.S.P.) Ribeirao Preto [41].

#### 2.7.2. Quantification of Fungal Membrane Sterols

For the quantification of fungal membrane sterols, five microliters of a 1 × 10^6^ conidia/mL suspension were placed in contact with sub-inhibitory concentrations of nonyl (7.8 mg/L), fluconazole (128 mg/L), and amphotericin B (1 mg/L). Untreated fungi were used as controls. The suspensions were incubated for 7 days at 37 °C. After this period, the tubes were centrifuged at 4 °C at 5000 rpm for 30 min; the supernatant was removed, and the dry weight was determined. Approximately 3 mL of a 25% KOH alcoholic solution was added to sterile glass tubes containing 1 g of dry weight and shaken for 1 min. The tubes were then placed in a water bath at 85 °C for 1 h and cooled to room temperature. After cooling, a mixture of 1 mL of sterile distilled water and 3 mL of n-heptane (Sigma-Aldrich) was added to the sterile glass beads. Vigorous vortexing was performed for 15 min. Supernatants were removed, placed in 1.5 mL microtubes, and stored at −20 °C overnight. The following day, the samples were read using an ultraviolet-visible spectrophotometer at a wavelength of 282 nm. A calibration curve was constructed using standard ergosterol (Sigma-Aldrich) [20,39,42].

#### 2.7.3. Confocal Laser Scanning Microscopy after Staining with Calcofluor White

For confocal microscopy, a 1 × 10^6^ cells/mL fungal suspension was treated with the same concentrations of nonyl, fluconazole, and amphotericin B in 24-well plates (Kasvi) containing sterile round coverslips. After 96 h of incubation, the plates were centrifuged, the culture medium was gently removed, and the wells were washed with PBS after successive centrifugation steps. A working solution of Calcofluor White (Sigma-Aldrich) was prepared at a concentration of 100 mg/L, and 30 µL of this solution was added to the wells. The plates were then reincubated at 37 °C for 45 min and protected from light. The wells were then washed with sterile PBS, and the plates were centrifuged. Coverslips were carefully removed and poured into 4 μL of Fluoromount-G (Sigma-Aldrich), previously deposited on microscope slides for observation under a confocal microscope, the ZEISS LSM 800 with Airyscan with image capture and processing software ZEN BLUE 2.3 System [20,43].

#### 2.7.4. Quantification of Reactive Oxygen Species (ROS)

Intracellular production of ROS before and after treatment of *T. rubrum* ATCC 28189 with nonyl was evaluated using 50 µM of 2,7 dichlorodihydrofluorescein diacetate (H_2_DCFDA, Invitrogen by Thermo Fisher Scientific, Waltham, MA, USA). Amphotericin B and hydrogen peroxide (10 mM) were used as controls. The same concentrations of the compounds as in Section 2.7.1 and Section 2.7.2 were used for treatment. All samples were incubated with H_2_DCFDA for 30 min at room temperature and subsequently analyzed on a BD FACSCanto I flow cytometer using a 530/30 nm wavelength filter for fluorescence detection. The data were analyzed using FlowJo 10.1 software [44,45].

#### 2.7.5. Apoptosis/Necrosis

The Annexin V-FITC apoptosis detection kit (A9210; Sigma-Aldrich) was used according to the manufacturer’s recommendations. To this, 1.5 mL of the fungal suspension at a final concentration of 1 × 10^6^ cells/mL was treated with nonyl (7.8 mg/L), amphothericin B (4 mg/L), or fluconazole (256 mg/L). The samples were analyzed using a BD FACSCanto I flow cytometer.

#### 2.7.6. Analysis of Differential Gene Expression of *T. rubrum* ATCC 28189 Treated with Nonyl by Real-Time PCR

The relative expression of genes related to ergosterol synthesis after treatment of *T. rubrum* with nonyl protocatechuate was evaluated using real-time PCR (qPCR) and compared to untreated fungal gene expression. Total RNA was extracted using the Illustra RNAspin Isolation Kit (G.E. Life Sciences) according to the manufacturer’s guidelines. The concentration and purity were determined by reading in a Nanodrop™ 2000 spectrophotometer (Thermo Scientific™, Waltham, MA, USA) at absorbances of 260 and 280 nm, and the integrity was evaluated by capillary electrophoresis using the Agilent 2100 Bioanalyzer equipment (Agilent Technologies, Palo Alto, CA, USA). Specific primers are represented in Table 1 [46,47,48]. Total RNA was treated with the DNase I kit (Sigma-Aldrich), and complementary DNA (cDNA) was synthesized using a high-capacity cDNA reverse transcription kit (Applied Biosystems), according to the manufacturer’s guidelines. The qPCRs were performed on the 7500 Real-Time PCR Instrument (Applied Biosystems by Thermo Fisher Scientific) of the School of Pharmaceutical Sciences of U.N.E.S.P. Araraquara with the Power SYBR™ Green PCR Master Mix detection system (Applied Biosystems by Thermo Fisher Scientific).

Reactions were prepared in a total volume of 20 µL, using 52 ng of cDNA and 0.5 µM of each primer. The relative expression was calculated by the 2^−∆∆Ct^ method, using the glyceraldehyde-3-phosphate dehydrogenase (GAPDH) and DNA-dependent RNA polymerase II (rpb2) genes as endogenous [46,49,50].

### 2.8. Statistical Analysis

All assays were performed in triplicate and in three independent experiments. Data were analyzed using an analysis of variance with Bonferroni’s post-hoc test. Data generated by qPCR were analyzed using the Student’s *t*-test. All data was generated using GraphPad Prism 5.0 software. Statistical significance was set at *p* less than 0.05.

## 3. Results

### 3.1. Susceptibilities of T. rubrum and T. mentagrophytes Planktonic and Biofilm Forms to Antifungal Drugs and Nonyl 3, 4-Dihydroxybenzoate

Table 2 shows a comparison of the metabolic activities of *T. rubrum* and *T. mentagrophytes* in the planktonic and biofilm forms after treatment with different drugs and nonyl. For all strains, planktonic cells were more susceptible to treatment than biofilm forms. The concentrations of fluconazole that reduced at least 50% of the metabolic activity (MIC_50_) of the planktonic cells of *T. rubrum* ATCC 28189, *T. rubrum* 143, *T. mentagrophytes* ATCC 11481, and *T. mentagrophytes* 66 were 128, 32, 2, and 16 mg/L, respectively. Griseofulvin reduced the metabolic activity by ≥50% at 256 mg/L for *T. rubrum* 143, 4 mg/L for *T. rubrum* ATCC 28189, and 1 mg/L for *T. mentagrophytes* ATCC 11481 and *T. mentagrophytes* 66. Terbinafine showed an inhibition at 0.015 mg/L for *T. mentagrophytes* ATCC 11481, *T. rubrum* ATCC 28189, and *T. rubrum* 143, and 0.06 mg/L for *T. mentagrophytes* at 0.125 mg/L 66. Nonyl reduced the metabolic activity to 7.8 mg/L for *T. mentagrophytes* ATCC 11481 and 15.6 mg/L for the other strains.

The mature biofilms of *T. rubrum* 143, *T. rubrum* ATCC 28189, *T. mentagrophytes* ATCC 11481, and *T. mentagrophytes* 66 exposed to fluconazole and griseofulvin exhibited metabolic activity values similar to those observed in the untreated controls, with SMIC_50_ > 512 mg/L (Table 2). Treatment with terbinafine significantly reduced the metabolic activity of *T. rubrum* 143 and *T. mentagrophytes* ATCC 11489 biofilms at concentrations of 0.5 and 16 mg/L, respectively. In contrast, for *T. rubrum* ATCC 28189 and *T. mentagrophytes* 66 biofilms, the SMIC_50_ values were greater than the highest concentration tested (>32 mg/L). Nonyl-treated biofilms obtained a significant reduction in metabolic activity at concentrations of 62.5 mg/L for *T. rubrum* species and 125 and 250 mg/L for *T. mentagrophytes* ATCC 11489 and *T. mentagrophytes* 66, respectively (Table 2). These results showed that the compound was more effective against biofilms than fluconazole and griseofulvin for all the strains tested.

### 3.2. Scanning Electron Microscopy of Treated Biofilms

**Figure 1:**
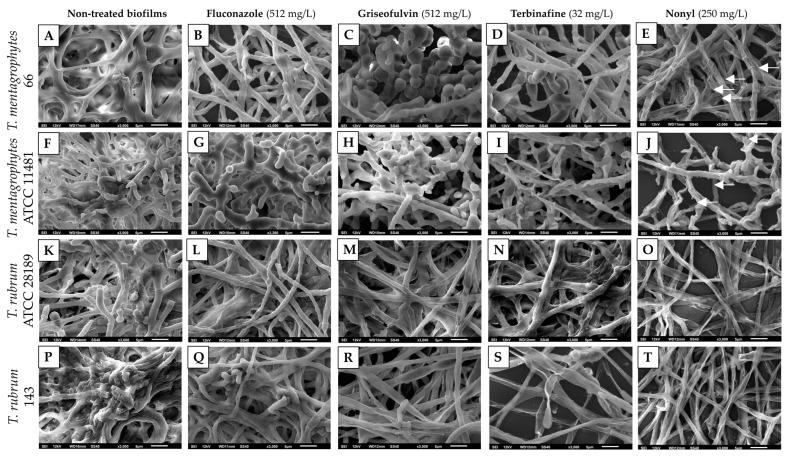
Scanning electron microscopy of *T. rubrum* and *T. mentagrophytes* mature biofilms, untreated (**A**,**F**,**K**,**P**) and treated with fluconazole (**B**,**G**,**L**,**Q**); griseofulvin (**C**,**H**,**M**,**R**); terbinafine (**D**,**I**,**N**,**S**) and nonyl 3,4-dihydroxybenzoate (**E**,**J**,**O**,**T**). White arrows denote small structures, similar to atrophied microconidia or extracellular vesicles (**E**,**J**).

SEM images showed that fluconazole and griseofulvin had minimal or no effect on *T. rubrum* biofilms (Figure 1L,M,Q,R). Terbinafine caused no apparent damage to *T. rubrum* ATCC 28189 hyphae (Figure 1N); however, visualization of *T. rubrum* 143 biofilms revealed the presence of destroyed hyphae and a reduction in the extracellular matrix (Figure 1S). Nonyl severely damaged *T. rubrum* ATCC 28189 (Figure 1O) and *T. rubrum* 143 (Figure 1T) biofilms, resulting in wilted hyphae, cell wall collapse, and a dramatic reduction in the extracellular matrix. However, few intact hyphae were observed in *T. rubrum* 143 (Figure 1J).

Concerning *T. mentagrophytes* biofilms, treatment with fluconazole (Figure 1B,G) and griseofulvin (Figure 1C,H) did not cause visible damage to the structures of the microbial communities. Moreover, griseofulvin stimulated the production of arthroconidia along the entire biofilm length. These structures, which are considered resistant, were observed especially in biofilms formed by *T. mentagrophytes* 66 (Figure 1C). Although terbinafine caused slight damage to *T. mentagrophytes* 66 biofilms (Figure 1D), severe damage was observed in the biofilms formed by the ATCC strain (Figure 1H). Nonyl caused significant damage to *T. mentagrophytes* 66 (Figure 1D) and *T. mentagrophytes* ATCC 11481 (Figure 1I) biofilms, causing leakage of cytoplasmic material and cell wall collapse. Interestingly, small round structures such as atrophied microconidia or perhaps extracellular vesicles (E.V.) were observed next to the damaged *T. mentagrophytes* hyphae treated with nonyl (white arrows in Figure 1D,I).

### 3.3. Confocal Microscopy of Treated Biofilms

**Figure 2:**
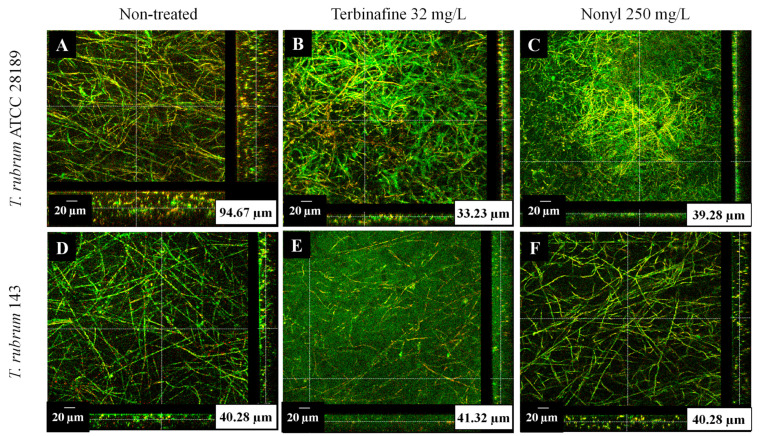

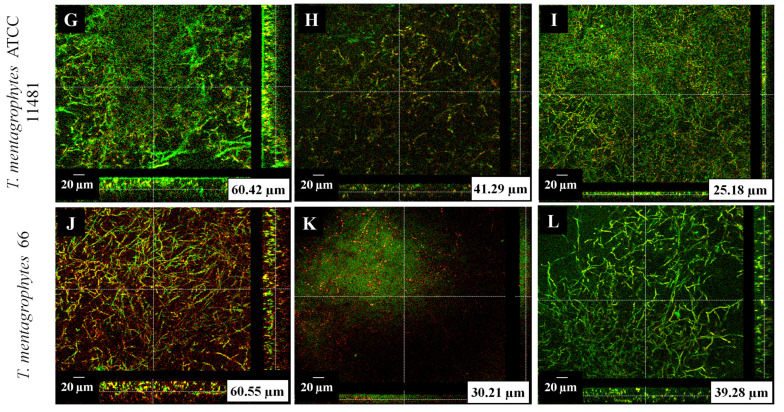
Orthogonal sections obtained by confocal laser scanning microscopy of mature *T. rubrum* and *T. mentagrophytes* biofilms, untreated (**A**,**D**,**G**,**J**); treated with terbinafine 32 mg/L (**B**,**E**,**H**,**K**) and treated with nonyl 3, 4-dihydroxybenzoate 250 mg/L (**C**,**F**,**I**,**L**), showed that both treatments reduced the thickness and/or mass of biofilms. Pictures were taken at 63× magnification, 20 µm bars. Metabolically active cells are stained red (FUN 1), while cells surrounded by polysaccharide material are stained green (ConA). The image has been adjusted for brightness.

Confocal microscopy of the biofilms treated with terbinafine and nonyl was performed to determine the thickness of the microbial communities after exposure to these compounds. The analysis showed that untreated *T. rubrum* ATCC 28189 and the clinical isolate 143 biofilms had an average thickness of 94.67 μm (Figure 2A) and 40.28 μm (Figure 2D), respectively. For the *T. rubrum* ATCC strain, treatment with terbinafine or nonyl significantly reduced the thickness of the biofilms on average to 33.23 µm (Figure 2B) and 39.28 µm (Figure 2C), respectively. There was no reduction in the thickness of biofilms of *T. rubrum* 143 treated with terbinafine (Figure 2E) or untreated (Figure 2F); however, in the latter, it was possible to observe a much less dense biofilm than in the control and terbinafine treatments. Untreated biofilms of *T. mentagrophytes* ATCC 11481 and the clinical isolate 66 showed an average thickness of 60.55 µm (Figure 2G) and 60.42 µm (Figure 2J). Treatment with terbinafine or nonyl significantly reduced the thickness of the biofilms formed by both *T. mentagrophytes* ATCC (Figure 2H,I) and the clinical isolate (Figure 2K,L).

### 3.4. Determination of the Mechanism of Action of Nonyl Protocatechuate

#### 3.4.1. Transmission Electron Microscopy (TEM)

**Figure 3:**
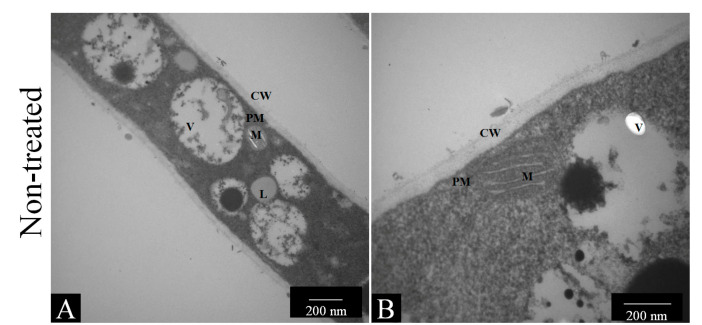

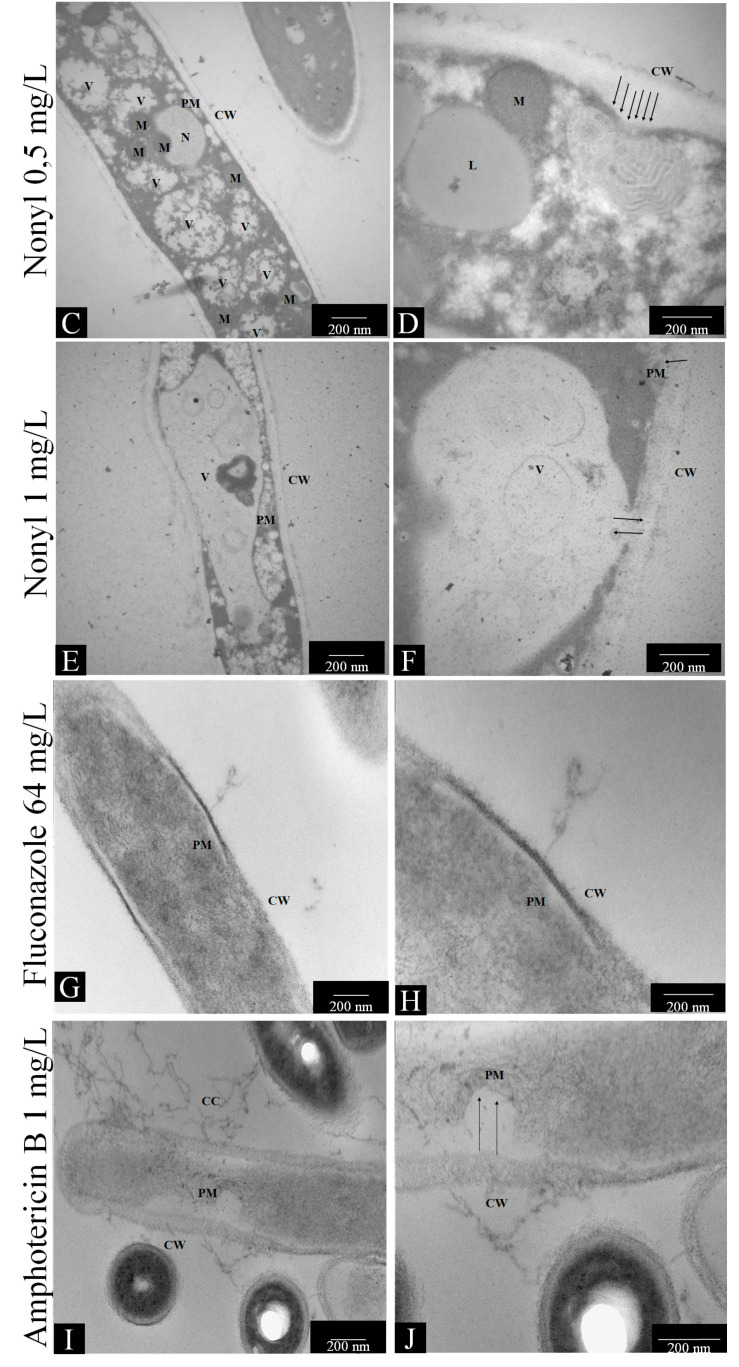
Transmission electron microscopy of *T. rubrum* 28189, untreated and treated with nonyl protocatechuate, fluconazole, and amphotericin B. (**A**) and (**B**) show untreated hyphae in the longitudinal section. It is possible to observe the cell wall (CW), the plasma membrane (PM), and some organelles such as mitochondria (M) and vacuoles (V), as well as some lipids present in the cytoplasm (CC: cytoplasm content). With treatment, there was a significant increase in vacuoles (**C**,**E**), disarrangement and/or disruption of the plasma membrane (PM) (**D**–**J**), and even disappearance of some organelles (**E**–**J**). The arrows denote a bulging or even rupture of the plasmatic membrane. Figures in panels (**A**,**C**,**E**,**G**,**I**) were taken at 20K× magnification, while figures in panels (**B**,**D**,**F**,**H**,**J**) were taken at 67K× magnification; bars were 200 nm.

Microscopic results showed that nonyl and the drugs caused significant changes in the hyphae of *T. rubrum* ATCC 28189 (Figure 3). Figure 3A,B show the longitudinal sections of untreated *T. rubrum* hyphae with intact cell walls, plasma membranes, and organelles. Treatment with nonyl at a concentration of 0.5 mg/L caused an increase in vacuoles and plasma membrane derangement in some areas (Figure 3C,D). Treatment with 1 mg/L of nonyl caused significant damage, with vacuoles covering almost the entire cytoplasm (Figure 3E). There was a possible rupture of the plasma membrane and an apparent osmotic imbalance (Figure 3F). However, there was no change in cell morphology, indicating that there was no significant change in the cell wall. Fluconazole treatment resulted in plasma membrane derangement (Figure 3G,H), while amphotericin B treatment resulted in visible pore formation and leakage of cytoplasmic content (Figure 3I,J).

#### 3.4.2. Quantification of Fungal Membrane Sterols

**Figure 4:**
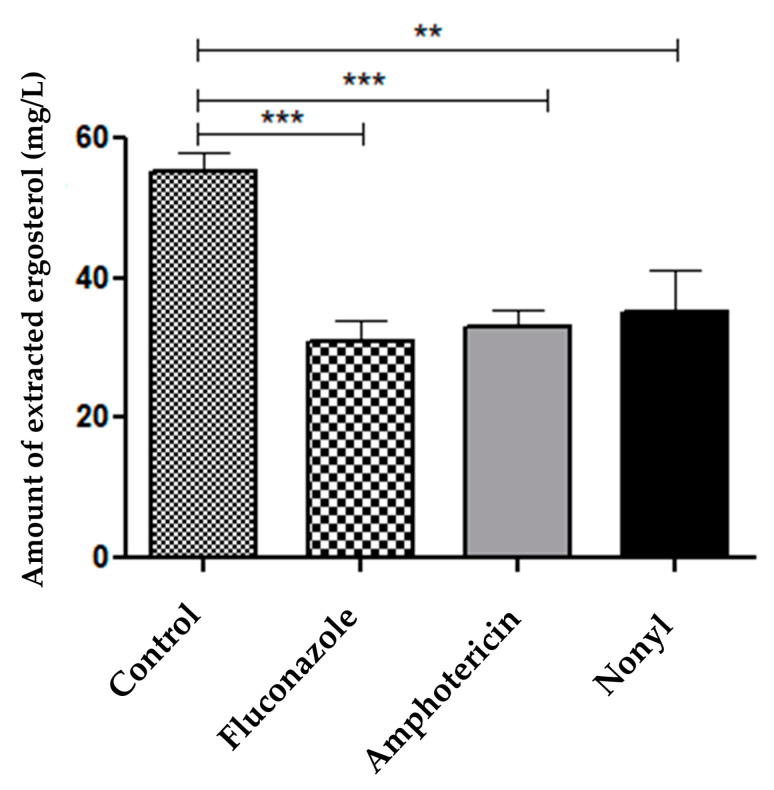
Levels of sterols extracted from the untreated control after treatment with nonyl (7.8 mg/L), fluconazole (128 mg/L), and amphotericin B (1 mg/L). ** *p* < 0.01; *** *p* < 0.001.

Sterol levels were reduced after treatment with nonyl (*p* < 0.01) and control antifungals (*p* < 0.001) (Figure 4).

#### 3.4.3. Verification of Cell Wall Morphology and Damage by Calcofluor White Staining and Confocal Microscopy

**Figure 5:**
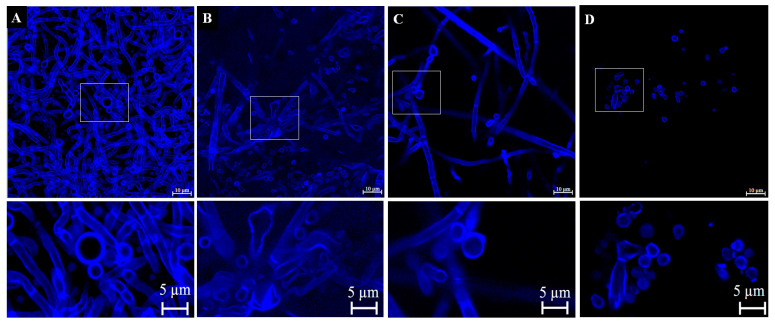
Confocal laser scanning microscopy images of untreated *T. rubrum* ATCC 28189 (**A**), treated with 7.8 mg/L nonyl (**B**), 128 mg/L fluconazole (**C**), and 1 mg/L amphotericin B (**D**) at a magnification of 100×; bars, 10 µm. The panels show cell walls stained with calcofluor white, even in those in which more extensive damage and inhibition of hyphal development occurred. Lower panels represent enlargements of the areas demarcated by rectangles for better visualization of the damage caused by the treatments to the fungi cell walls at the magnification of 255×; bars, 5 µm.

The results in Figure 5 show that in the untreated control, the morphology was intact, with cell walls well-demarcated by calcofluor (Figure 5A). Conformational changes were observed in the cells treated with nonyl (Figure 5B), fluconazole (Figure 5C), and amphotericin B (Figure 5D), which showed irregular shapes. Despite the damage caused by the nonyl groups, there was no impairment of the cell wall structure. Calcofluor is a non-specific fluorophore that binds to cell wall chitin, mainly in the polysaccharides β 1, 3, and β 1,4 [51].

#### 3.4.4. ROS Quantification

**Figure 6:**
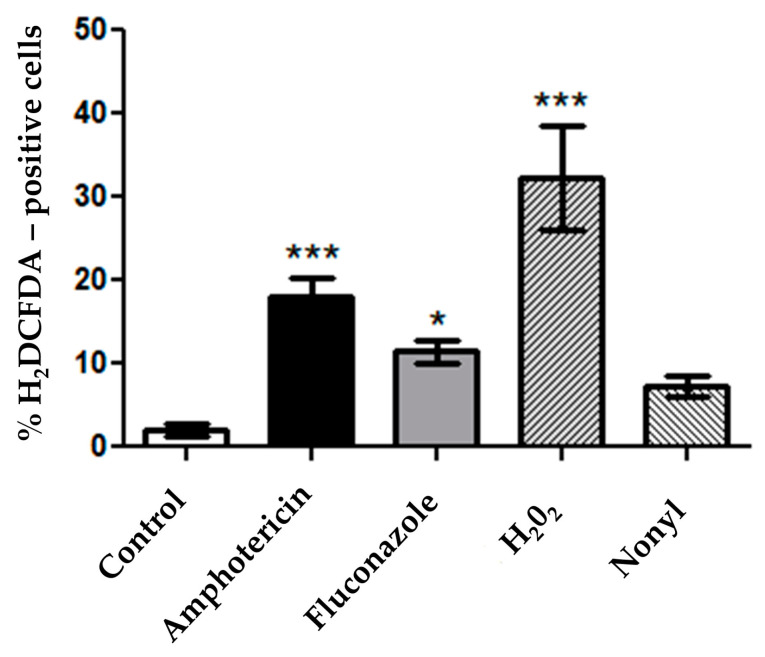
Measurement of ROS production without and after treatment with amphotericin B (AMB), hydrogen peroxide (H_2_O_2_), and nonyl protocatechuate. Three independent experiments were performed in triplicate, and the results are expressed as the mean ± the standard deviation of the percentage of positive cells. * *p* < 0.05; *** *p* <0.001; H_2_O_2_: hydrogen peroxide.

Intracellular quantification of ROS showed that nonyl treatment did not induce the production of reactive oxygen species when compared to the untreated control (*p* > 0.05) (Figure 6). The control treatment with hydrogen peroxide best induced the production of ROS compared to the other treatments (*p* < 0.001).

#### 3.4.5. Determination of the Mechanisms of Cell Death by Apoptosis/Necrosis

**Figure 7:**
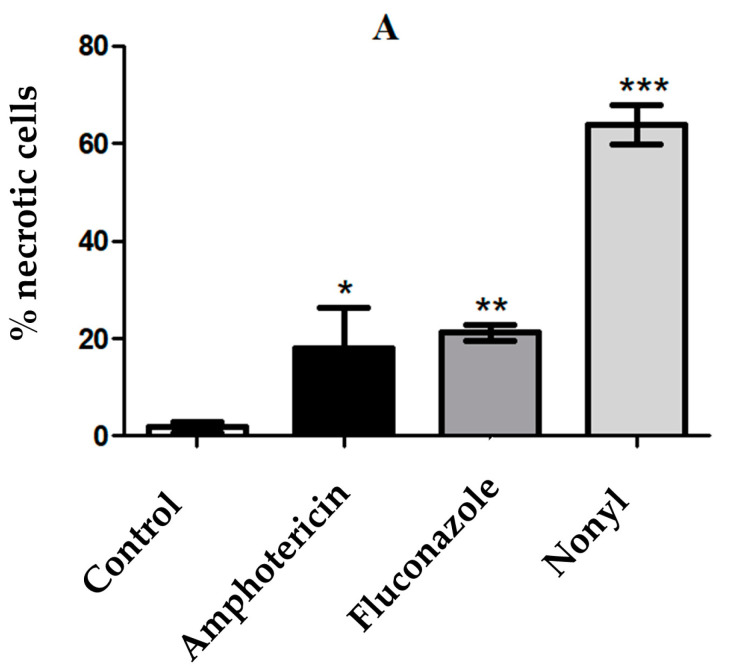

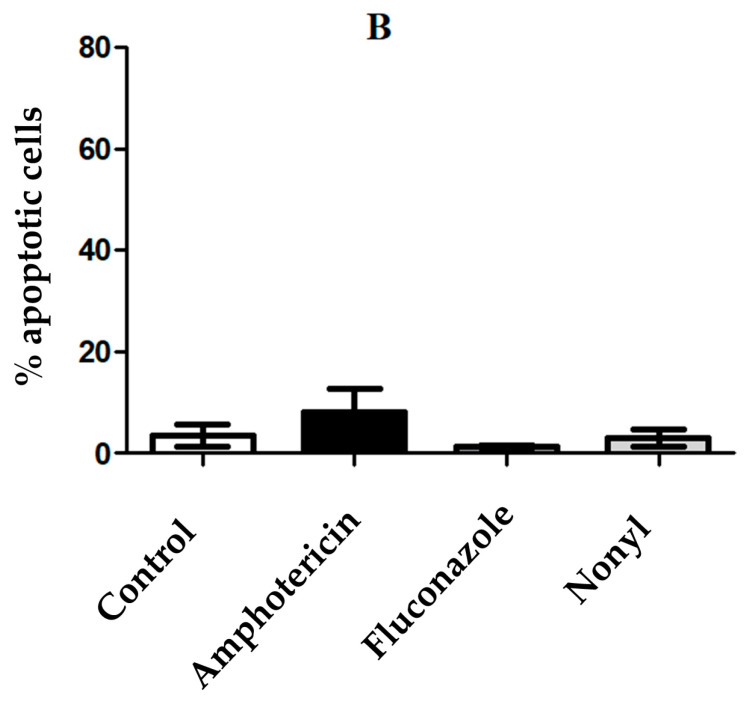
Mechanism of cell death by necrosis (**A**) and apoptosis (**B**) induced after treatment of *T. rubrum* ATCC 28189 with nonyl, fluconazole, and amphotericin B. Both significantly induced necrotic death (*p* < 0.05). * *p* < 0.05; ** *p* <0.01; *** *p* <0.001.

Nonyl, fluconazole, and amphotericin B significantly induced necrotic cell death compared to that in the untreated control (*p* < 0.05). Among the treated groups, the most significant induction was caused by the protocatechuic acid derivative (63%) (*p* < 0.001) (Figure 7).

#### 3.4.6. Analysis of Differential Gene Expression of *T. rubrum* ATCC MYA-4438 Treated with Nonyl Protocatechuate by Real-Time PCR

**Figure 8:**
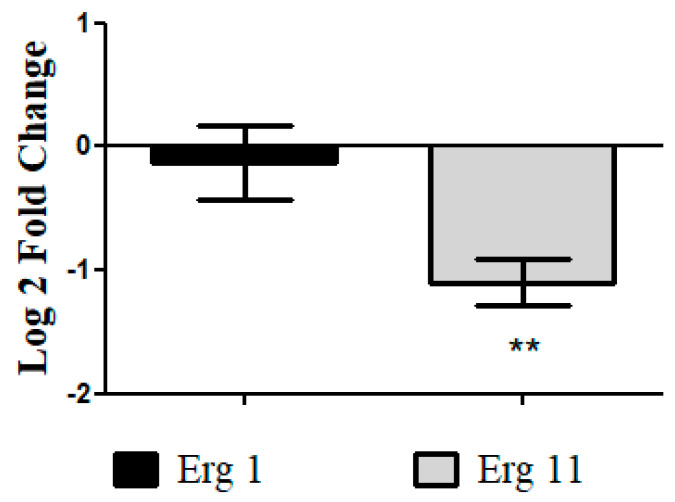
Relative expression levels of Erg 1 and Erg 11 genes represented in Log2Fold change in *T. rubrum* ATCC 28189 strain treated with nonyl protocatechuate, compared to the untreated control. Results from three independent experiments are expressed as the mean ± standard deviation. Statistical significance was determined by the Student’s *t*-test and indicated by asterisks: ** *p* < 0.01.

The relative expression results showed a negative modulation of Erg11 (*p* < 0.01) after treatment with nonyl (Figure 8), proving the membranolytic action of the protocatechuic acid derivative. Erg 11 encodes the enzyme lanosterol 14 α-demethylase, the main target of azole antifungals [47]. Gene modulation of Erg1 was insignificant, indicating that this compound does not act in the initial pathway of ergosterol biosynthesis (squalene epoxidase), similar to terbinafine.

## 4. Discussion

In this study, the susceptibilities of *T. rubrum* and *T. mentagrophytes* to the antifungal drugs fluconazole, griseofulvin, terbinafine, and a compound derivative of protocatechuic acid were tested under biofilm and planktonic conditions. Nonyl caused significant damage to the biofilms formed by all strains at a 62.5 mg/L concentration. Similar damage was observed in terbinafine-treated biofilms in *T. mentagrophytes* ATCC 11481 and *T. rubrum* 143. Furthermore, it is noteworthy that mature dermatophyte biofilms were resistant to all concentrations of fluconazole and griseofulvin. Although treatment with terbinafine damaged the topography of some biofilms and decreased their metabolic activity and thickness, many intact cells and a reasonable amount of extracellular matrix were still observed. Furthermore, it was necessary to apply terbinafine concentrations higher than 32 mg/L to eradicate biofilms from the other two strains, which is therapeutically problematic [52,53,54]. Variability in the strain-dependent resistance of dermatophyte biofilms is evident in the literature [4,7,20,38,55,56,57], which reinforces the need for increased testing to assist in the treatment of infections involving fungal communities.

Regarding griseofulvin in the SEM images, *T. mentagrophytes* biofilms treated with this drug produce structures such as arthroconidia throughout their length. Arthroconidia are resistant to certain antifungal drugs and adverse environmental conditions [58]. *T. mentagrophytes* spores remain viable after exposure to different concentrations of antifungal drugs such as griseofulvin [59].

Our SEM results also showed that treatment with nonyl in *T. rubrum* and *T. mentagrophytes* caused significant damage to the biofilms, resulting in the leakage of cytoplasmic contents and cell wall collapse. However, the antimicrobial effect of compounds derived from phenolic acids increases with an increasing number of alkyl side chains [30,60], which may explain the potency of nonyl 3,4-dihydroxybenzoate, which has nine carbons in the side alkyl chain. According to Kubo et al. [32], the hydrophilic portion of phenolic compounds can bind to the hydrophilic portion of the cell membrane, enabling it to enter and disrupt the lipid bilayer. Regarding *T. mentagrophytes* biofilms treated with the compound, in addition to the destruction of hyphae, we observed small round structures that remained intact after treatment, similar in size and morphology to extracellular vesicles (E.V.) that may be secreted after the stress caused by treatment of the biofilm. Previous studies have reported the presence of E.V. in the culture supernatants of *C. albicans* [61,62], *C. neoformans* [63], *H. capsulatum* [64], *P. brasiliensis* [65]*, Malassezia sympodialis* [66], and *T. interdigitale* [67]. In fungal infections, secretory mechanisms play a key role in virulence, and the identification of molecules associated with these vesicles and secretions is crucial [62,68].

Microorganisms in biofilm form may resist antimicrobials at concentrations up to 5000 times higher than those of drugs required to eradicate planktonic or free forms of the same microorganism [24,25,29,69]. Thus, there is an urgent need to acquire new knowledge about the pathophysiology of fungal biofilms to aid the search for new antimicrobial molecules with anti-biofilm activity [24,25,70]. The current study confirmed the resistance of biofilms formed by the two main dermatophyte species to some of the most commonly used antifungal agents worldwide, especially in developing countries, where these mycoses are most prevalent. In addition, it highlights the potent activity of a synthetic compound originating from protocatechuic acid, which can be isolated from several plants and vegetables and is commercially available.

Regarding the mechanism of action, nonyl caused significant changes in the ultrastructure of the hyphae, an effect similar to that observed for antifungals of the azole and allylamine classes, which act on the biosynthesis of ergosterol, damaging the cell membrane [71]. TEM electron microscopy showed that the compound binds directly to non-sterol lipids in the fungal cell membrane, similar to the polyene class, leading to increased wall permeability and subsequent membrane lysis. AMB, the main drug in the polyene class, forms channels in the fungal cell membrane that allow ions and organic compounds from the cytoplasm to escape, thus destroying the membrane’s function [45,72,73].

Fungal membranes are composed of several lipids, the most abundant of which is ergosterol. The results showed that treating dermatophytes with nonyl, fluconazole, and amphotericin B reduced the ergosterol levels. Azoles inhibit the cytochrome P450-dependent enzyme system responsible for the conversion of lanosterol to ergosterol [74]. In contrast, AMB binds to ergosterol, creating pores that cause osmotic imbalance. Recent hypotheses have shown that this drug has a “sponge” effect, making it capable of extracting ergosterol from membranes. [75,76,77]. Vaso et al. [39] quantified ergosterol in the fungal cell membrane and found lower amounts of ergosterol in both azoles and polyenes.

Chitin is the leading cell wall polysaccharide, formed mainly by β-1,3 and β-1,4 glucans. Calcofluor white is a nonspecific fluorophore that binds to these components and emits fluorescence when excited [51,78]. Our results revealed that the nonyl group caused irregularities in the hyphal morphology and rupture of the fungal membrane, as observed by TEM, leading to an osmotic imbalance and irregular morphology. After treatment with fluconazole, there was no damage to the cell wall; after treatment with AMB, there was a reduction in the number of cells and inhibition of hyphal formation. Bila et al. [20] observed that for dermatophytes, fluconazole did not cause damage to cells, corroborating the results of the present study.

The nonyl treatment did not induce significant ROS production compared to the untreated control. When compared to the controls, treatment with hydrogen peroxide was the one that best induced ROS production, followed by AMB and fluconazole. Singulani et al. [79] obtained similar results for *C. neoformans*, and Bila et al. [20] obtained similar results for dermatophytes. The authors showed that AMB induced ROS production. This secondary mechanism of action leads to cell death via oxidative stress [45,80,81]. Vandenbosch et al. [82] showed that strains of *Candida albicans* treated with miconazole and ketoconazole induced ROS production.

Nonyl protocatechuate induces necrotic cell death in more than 60% of cells. These results prove that this compound has a cell membrane as its primary target and has an amphiphilic nature, which breaks the plasma membrane and alters its fluidity and integrity, mainly because of its role in ergosterol biosynthesis. In cell death assays, annexin V, which specifically binds to phosphatidylserine, is widely used to detect the affinity of phosphatidylserine for the outer bilayer of the plasma membrane at the beginning of the apoptotic process. During necrosis and late apoptosis, there is an influx of propidium iodide, which stains these cells [79,83]. Apoptosis is programmed cell death associated with cellular homeostasis. The characteristic changes in apoptotic cells include ROS accumulation, DNA breaks, caspase activation, and phosphatidylserine externalization [83]. Necrosis is death resulting from direct cell injury and is defined as the swelling and lysis of cells and organelles [84,85].

Finally, in gene expression analysis, negative modulation of Erg11 was confirmed after treatment with nonyl, demonstrating the membranolytic action of the protocatechuic acid derivative. Erg 11 encodes the enzyme lanosterol 14 α-demethylase, the main target of azole antifungals [47]. Gene modulation of Erg1 was insignificant, indicating that this compound does not act in the initial pathway of ergosterol biosynthesis (squalene epoxidase), similar to terbinafine.

## 5. Conclusions

This work demonstrated the effective anti-biofilm activity of the compound nonyl 3,4-dihydroxybenzoate against different strains of *T. rubrum* and *T. mentagrophytes* and showed that nonyl activity was better than the tested antifungal drugs. Various biochemical and molecular assays have demonstrated membranolytic activity in fungal cells. These findings must be considered because onychomycosis is a mycosis that is difficult, time-consuming, and expensive to treat, with high rates of relapse and treatment abandonment by a large part of the population. Furthermore, these results pave the way for new studies that enable this compound to evolve in the antifungal pipeline and become a drug candidate.

## Figures and Tables

**Table 1:** Oligonucleotide primers were used to evaluate the relative gene expression of *T. rubrum* ATCC 28189, treated with nonyl protocatechuate, and compared to the untreated fungus.

Gene	5′-3′ Oligonucleotides	Efficiency
Gapdh	F: GCGTGACCCAGCCAACA R: CGGTGGACTCGACGATGTAGT	90.7
Rpb2—DNA-dependent RNA polymerase II	F: TACAGGAAATTGGGGTGAGC R: GTTCGTCGAAGATGGGAAAG	104.1
ERG1	F: GTGAAGATACCTTTCCCTAGCG R: TTATGGTAGAAACGGCCTTGG	98.41
ERG 11	F: CACTTCCTTGCCCTGTAGAGATC R: GGAGTTTTCAATGTCAGCAAGGTTT	98.85

F: foward; R: reverse.

**Table 2:** Comparison of planktonic cells and biofilms’ susceptibilities to antifungal drugs and the compound nonyl 3, 4-dihydroxybenzoate.

Compound/Drug	Phenotype	TM 66	TM ATCC 11489	TR ATCC 28189	TR 143
Fluconazole	Planktonic (MIC_50_)	16	2	128	32
	Biofilm (SMIC_50_)	>512	>512	>512	>512
Griseofulvin	Planktonic (MIC_50_)	1	1	4	256
	Biofilm (SMIC_50_)	>512	>512	>512	>512
Terbinafine	Planktonic (MIC_50_)	0.06	0.015	0.125	0.125
	Biofilm (SMIC_50_)	>32	16	>32	0.5
Nonyl 3, 4-dihydroxybenzoate	Planktonic (MIC_50_)	15.6	7.8	15.6	15.6
	Biofilm (SMIC_50_)	250	125	62.5	62.5

TM, *Trichophyton mentagrophytes*; TR, *Trichophyton rubrum*; MIC_50_: minimal inhibitory concentration 50%; SMIC_50_: sessile minimal inhibitory concentration.

## Data Availability

Not applicable.

## Supplementary Materials

The following supporting information can be downloaded at: https://www.mdpi.com/article/10.3390/pharmaceutics15051402/s1, Figure S1. ^1^H NMR spectrum of nonyl protocatechuate (300 MHz, CDCl_3_). Figure S2. ^13^C NMR spectrum of nonyl protocatechuate (75 MHz, DMSO-*d*_6_). Figure S3. HPLC-PAD chromatogram [MeOH:H_2_O (98:2); 254 nm].
