# Supplementary material for: Membranolytic Activity Profile of Nonyl 3,4-Dihydroxybenzoate: A New Anti-Biofilm Compound for the Treatment of Dermatophytosis
Source: Pharmaceutics. 2023 May 4;15(5):1402. doi: 10.3390/pharmaceutics15051402 (PMC10223064; doi:10.3390/pharmaceutics15051402)
Supplement: Supplementary file 1 [file pharmaceutics-15-01402-s001.zip › pharmaceutics-2089944-supplementary.pdf]

Figure S1. ^{1}H NMR spectrum of nonyl protocatechuate (300 MHz, $CDCl_3$)

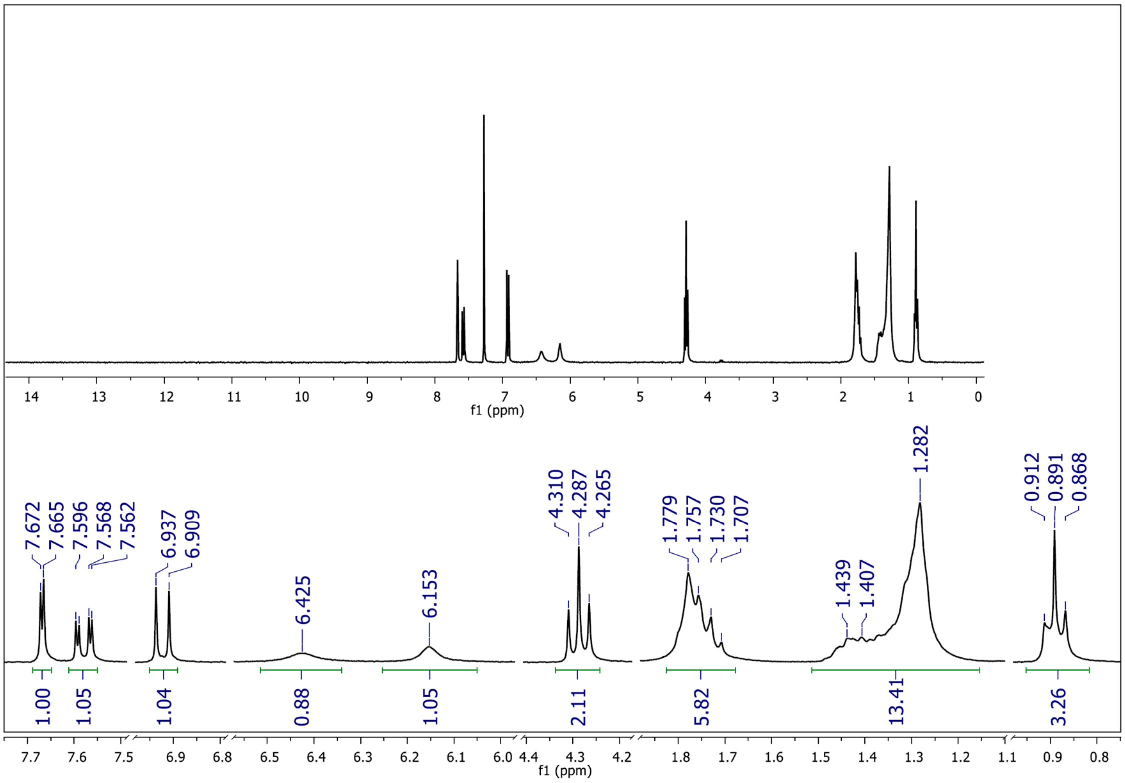

Figure S2. ^{13}C NMR spectrum of nonyl protocatechuate (75 MHz, DMSO-d_6)

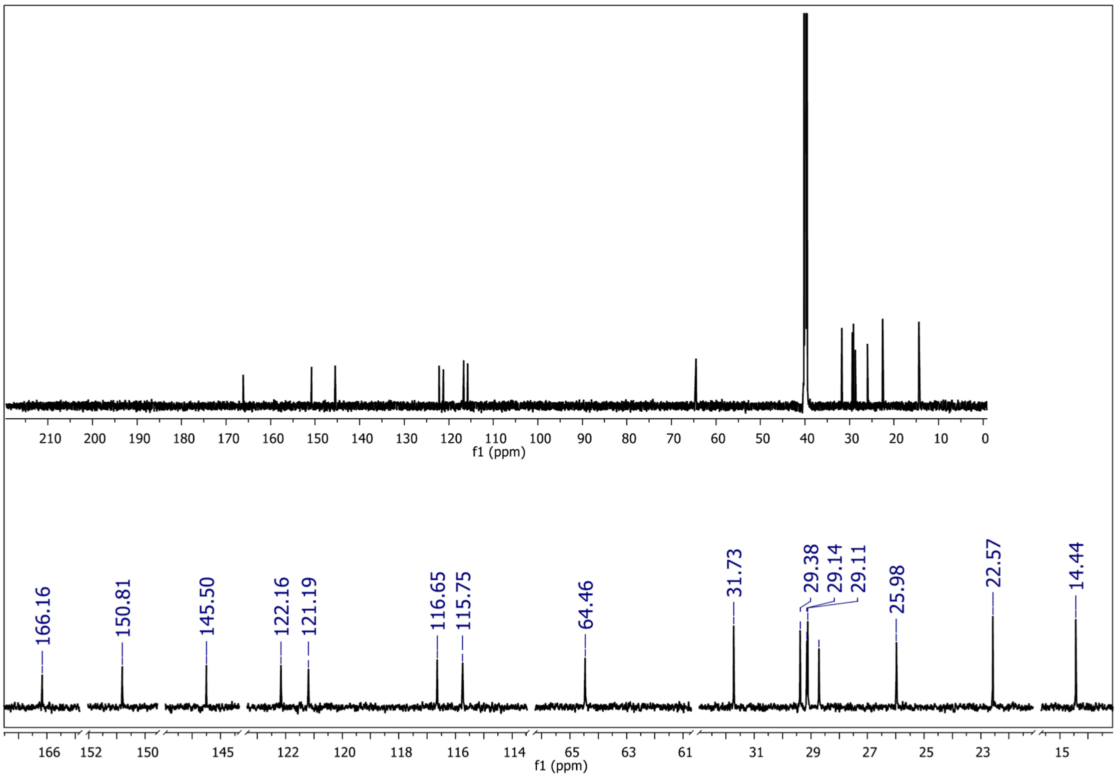

Figure S3. HPLC-PAD chromatogram [MeOH:H_2O (98:2); 254 nm]

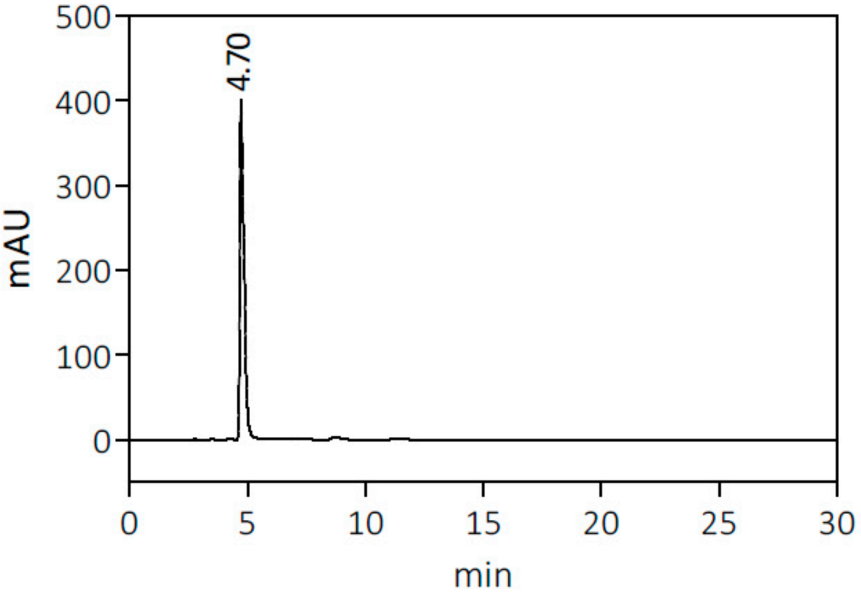

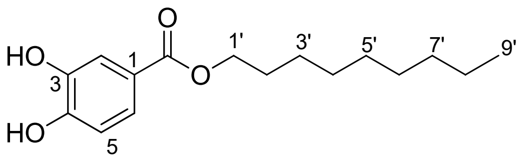

^{1}H NMR (300 MHz, $CDCl_3$) *δ* in ppm (multiplicity; *J* in Hz): 7.67 (d, 2.1, H-2), 7.58 (dd, 8.4 and 2.1, H-6), 6.92 (d, 8.4, H-5), 6.42 (br s; 4-OH), 6.15 (s; 3-OH), 4.29 (t, 6.7, H-1'), 1,71–1.78 (H-2'and H-3'), 1.28–1.44 (H-4'–H-8'), 0.89 (t, 6.7, H-9').

^{13}C NMR (75 MHz, DMSO-d_6) *δ* in ppm: 166.2 (C-7), 150.8 (C-4), 145.5 (C-3), 122.2 (C-6), 121.2 (C-1), 116.6 (C-5), 115.7 (C-2), 64.5 (C-1'), 31.7 (C-2'), 29.4, 29.2, 29.1, 28.7, 26.0 and 22.6 (C-3'–C8'), 14.1 (C-9').

Purity: 98% (at 254 nm).
